# Impacts of accelerating deployment of offshore windfarms on near-surface climate

**DOI:** 10.1038/s41598-022-22868-9

**Published:** 2022-10-31

**Authors:** Naveed Akhtar, Beate Geyer, Corinna Schrum

**Affiliations:** 1 Institute of Coastal Systems - Analysis and Modeling, Helmholtz-Zentrum Hereon, Geesthacht, Germany; 2 Center for Earth System Research and Sustainability, Institute of Oceanography, Universität Hamburg, Hamburg, Germany

**Keywords:** Environmental impact, Physical oceanography

## Abstract

The European Union has set the ambitious goal of becoming climate neutral by 2050, which has stimulated renewable energy production and accelerated the deployment of offshore wind energy in the North Sea. Here, a high-resolution regional climate model was used to investigate the impact on the sea surface climate of large-scale offshore wind farms that are proposed for the North Sea. The results show a significant reduction in the air-sea heat fluxes and a local, annual mean net cooling of the lower atmosphere in the wind farm areas down to more than 2.0 Wm^−2^, due to a decrease in 10 m wind speed and turbulent kinetic energy and an increase in low-level clouds. Mean surface winds decreased by approximately 1 ms^−1^ downstream of wind farms. Furthermore, an increase of approximately 5% in mean precipitation was found over the wind farm areas. At a seasonal timescale, these differences are higher during winter and autumn than in other seasons. Although the offshore wind farms reduce the heat transport from the ocean to the atmosphere in the region of large wind farms, the atmospheric layers below the hub height show an increase in temperature, which is on the order of up to 10% of the climate change signal at the end of the century, but it is much smaller than the interannual climate variability. In contrast, wind speed changes are larger than projected mean wind speed changes due to climate change. Our results suggest that the impacts of large clustered offshore wind farms should be considered in climate change impact studies. Moreover, the identified offshore windfarm impacts on the sea surface climate and the introduced spatial pattern in atmospheric conditions, in particular the modeled wind speed changes, suggest potential impacts on local ocean dynamics and the structure of the marine ecosystem. This should be considered in future scenarios for the North Sea marine environment and taken into account as a structuring influence in the offshore environment.

## Introduction

To reduce carbon emissions and pursue efforts to keep the temperature rise below 1.5 °C, the European Commission aims to increase green energy, with one of the most important contributions expected to arise from offshore wind energy. The latest report indicates that the European Union (EU) aims to install 300 GW of offshore wind energy by 2050. If the targets of the United Kingdom and Norway are added, this figure rises to 400 GW^1^. Approximately 47% of this amount will be installed in the North Sea^2^, as the wind resources in the North Sea are stronger and more reliable at shallow water depths compared to other European Seas. This has made the North Sea one of the hotspots for offshore wind farm (OWF) development, with the massive deployment of sizable OWFs clustered in close proximity to each other (see Fig. 1 of Akhtar et al. 2021^3^). Wind farms are usually clustered around transmission lines to minimize deployment and operation costs.

**Figure 1:**
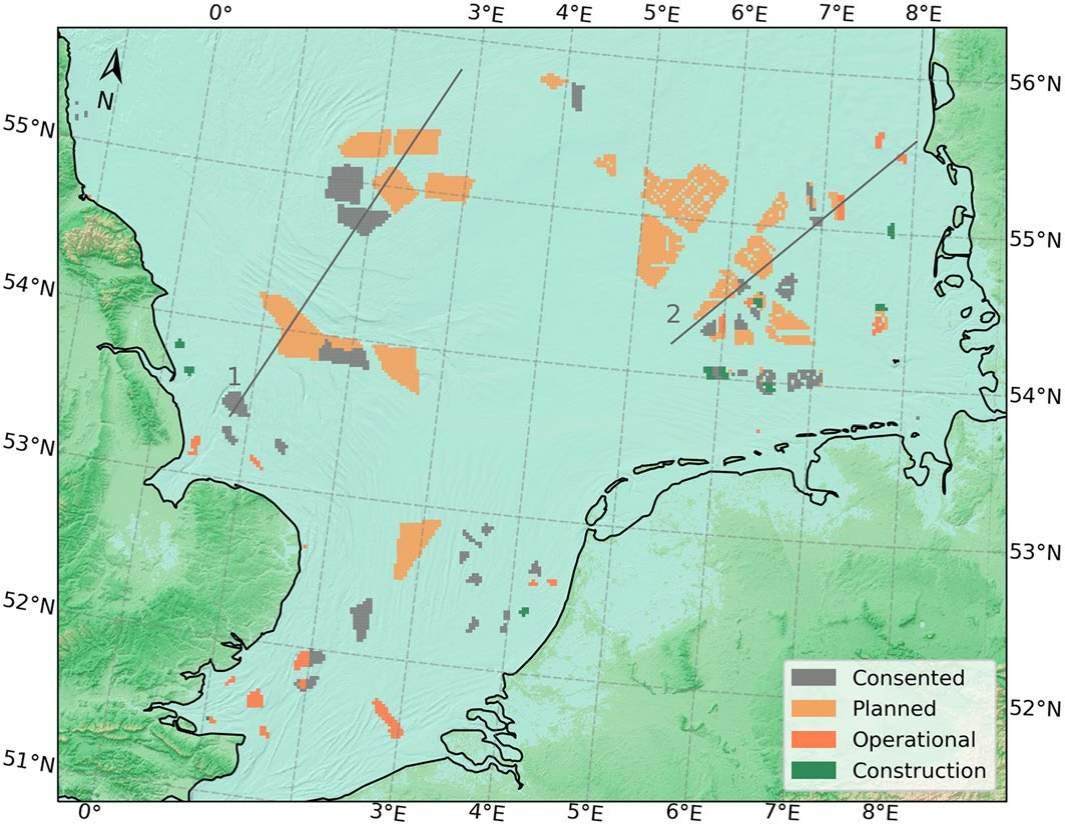
Scenario configuration for OWFs used for model scenario-based OWFs in the North Sea^1^. Colored polygons indicate the planning status of the OWFs by 2015 in the North Sea and the land-sea mask of the model domain. Gray lines indicate the transects used for analysis. This figure was created with Matplotlib (Hunter, J. D., Matplotlib: a 2D graphics environment. Computing in Science and Engineering 9, 2007) and Cartopy (Met office, Cartopy: a cartographic python library with a matplotlib interface. Exeter, Devon, https://scitools.org.uk/cartopy, 2015).

In an earlier article^3^, how the future deployment of offshore wind farm capacities at the planned scale in the North Sea could significantly affect power generation downstream of wind farms was investigated, which could lead to increasing costs of power generation. The present study aims to investigate the impact of such large-scale, clustered offshore wind farms on near-surface atmospheric conditions and air-sea fluxes.

Wind turbines extract kinetic energy (KE) from the mean atmospheric flow and convert a part of it into electric energy and the remaining part into turbulent kinetic energy (TKE) that drives wakes and a downwind wind speed deficit^4–7^. Additionally, the interaction of wind and wind turbines induces wind shear, which increases the TKE in the wake downwind. The KE extracted by wind turbines is replenished by the acceleration of air masses within the boundary layer due to large-scale pressure forces, which become unbalanced by apparent Coriolis forces due to the extraction of wind speed by wind turbines^8^.

The wakes generated by wind farms are expected to be longer over the ocean than over land due to the weaker turbulence intensity over the ocean^9,10^. Both numerical^5,11–13^ and analytical^10,14^ studies have predicted these prolonged OWF wakes. Observations based on satellite data^15^ and recent in situ aircraft measurements^16^ also confirmed these long wakes. The extent of the wake strongly depends on the atmospheric conditions; in stably stratified atmospheric conditions, wakes generated by wind farms can extend up to 50—70 km at hub height^16,17^.

Observational evidence shows that the wakes generated by wind turbines affect the whole structure of the planetary boundary layer and modify the near-surface local flow^18–20^. A field campaign measurement indicated that the presence of an offshore wind turbine reduces the near-surface TKE and that wind speed induces warming during stable and neutral conditions and cooing during unstable conditions^21^. Remote sensing observations indicate a net warming of approximately 0.7 °C in areas that are densely covered with onshore wind farms^18,22^. Experiments based on wind tunnels have suggested that wakes generated by wind farms can modify the energy budget^23^. Studies based on image analysis indicate fog formation and dispersion over OWFs due to increased mixing downwind of wind farms^14,24^. A decrease in net upward sensible heat flux due to enhanced vertical mixing is reported downwind of OWFs during stably stratified atmospheric conditions^25^. Recent in situ airborne measurements show that wakes can modify the temperature and humidity in the order of 0.5 K and 0.5 g kg^−1^, even 60 km downwind of the OWFs^13^. Changes in temperature and moisture at the hub height are associated with changes in temperature and moisture at the surface, which in turn modify surface turbulent fluxes^12,13^. During airborne measurements, cloud formation above wind farms has also been observed under slightly stable conditions and nearly saturated relative humidity^12^.

Process studies based on numerical simulations show that wind farms strongly modify the surface temperature, heat fluxes, TKE, and wind patterns^26,27^. The aforementioned studies set the wind farms as a drag at the model’s lowest level by increasing the aerodynamic roughness length or drag coefficient. However, such an approach induces a strong increase in the vertical fluxes of humidity^28^. In contrast, more localized and minimal impacts at both regional and global scales are found on temperature, heat fluxes, clouds, and precipitation when wind turbines are represented as momentum sinks and sources of TKE^29^. Additionally, the latter approach agrees well with the large-scale eddy simulation models^5,28–30^. Studies using wind turbines as momentum sinks and sources of TKE indicate that wind turbines influence atmospheric stratification by changing the surface temperature by approximately 1 °C for onshore installation, particularly during nocturnal stable conditions, due to the mixing of warmer air toward the surface^4^. Numerical simulations also found small increases in temperature and precipitation amounts over the OWFs^31,32^. Due to enhanced vertical mixing in the rotor area—drying and heating near the surface and moistening and cooling aloft—wind farms can alter the local climate by changing the energy budget^12^. Changes in the wind speed and TKE can impact the turbulent flux (that is, a decrease in sensible heat flux and an increase in latent flux)^12^. However, these results are based on high-resolution regional climate model simulations that were performed only for a very short time period (one day); therefore, the question remains as to whether OWFs alter the near mean surface wind field and mean sea surface heat fluxes and hence impact the regional climate. Furthermore, the direction of the impact (i.e., increase or decrease) in turbulent fluxes is still under discussion. For example, a study based on a high-resolution large-eddy simulation (LES) of stable boundary layer conditions reported a reduction of approximately 15% in heat fluxes over large wind farms^33^. In contrast, another study based on an LES found an increase of approximately 10—15% in the surface heat flux^34^. Similarly, studies with simulated typical weather conditions occurring in the summer season based on the mesoscale climate model METRAS^32^ reported a decrease in surface fluxes. In contrast, a case study using WRF^12^ showed an increase in the surface fluxes, but their results were based on a simulation that was only one day long. Therefore, it is important to analyze how the sea surface fluxes change after the deployment of large OWFs in the North Sea in the long term through continuous simulations that include all weather conditions.

A change in the turbulent and/or radiative fluxes can directly modify the energy budget of the atmosphere^35,36^. Anomalies in net sea surface heat flux play an important role in the local climate^37,38^, and a better estimate is crucial for accurate modeling of the regional climate, ocean dynamics and ecosystem of the North Sea. Here, the impact of existing and planned OWFs (Fig. 1) on sea surface fluxes and other important atmospheric variables in the North Sea using a regional climate model was analyzed. The aim was to quantify the impact of future large-scale offshore wind energy production and to assess whether climate mitigation measures, such as large-scale offshore energy production, need to be considered in regional climate and climate impact scenarios for the marine atmosphere and marine hydro and ecosystem dynamics.

## Experimental design

In the present study, the nonhydrostatic regional climate model COSMO-CLM^39^ was employed to simulate the impact of wind farms on the local atmospheric dynamics, spatial–temporal pattern of wind speed, and sea surface fluxes for an upcoming wind farm scenario in the North Sea (as shown in Fig. 1). A wind farm parameterization^5^ has been implemented in COSMO-CLM^30,40^ to consider the effects of wind farms. This wind farm parameterization represents the wind turbines as a sink of KE and source of TKE in each layer intersecting the rotor area. The wind turbines convert a part of the extracted KE into electric power, whereas the remaining part is converted into TKE. The amount of the extracted KE depends on the wind speed, air density, density of the wind turbines, rotor diameter, thrust, and power coefficients^41^. The thrust and power coefficients are a function of wind speed and are derived from the theoretical National Renewable Energy Laboratory (NREL) 5 MW reference wind turbine for offshore system development^41^. The data from the NREL 5 MW turbine are originally derived from the REPower 5 MW offshore wind turbine. The wind turbines have cut-in and cut-out wind speeds of 3 ms^−1^ and 25 ms^−1^, respectively, whereas the rated power is reached at 11.4 ms^−1^. It is important to mention that due to technological advancement, the sizes of the wind turbines are rapidly changing in the North Sea. Here, wind turbines with a hub height of 90 m and a rotor diameter of 126 m are used that fall well within the range of operating wind farms^42^. The use of 5 MW turbines in the present simulation can be considered a reference for further scenario simulations with higher hub heights and larger rotor areas. In this experiment, multiple wind turbines are contained within a grid box with a turbine density of approximately 1.8 × 10^–6^ m^−2^. Due to the relatively coarse resolution of the RCM, which is approximately 2 km in this experiment, the wake effects of individual wind turbines are not resolved. The average effect of the wind turbines within the grid box is estimated using the average grid box velocity. For more details on the wind farm parameterization and its implementation, the reader is referred to previous studies^29,30,43^.

COSMO-CLM uses a rotated horizontal grid with a spacing of 0.02° (~ 2 km; 396 × 436 grid cells) and 62 vertical levels with 5 levels within the rotor area. A numerical time step of 12 s with a third-order Runge–Kutta numerical integration scheme is used in the experiment. Over the sea, the roughness length is computed using the Charnock formula^44^. Physical options included a delta-two-stream scheme for short and longwave radiation, a cloud microphysics scheme, and a one-dimensional prognostic TKE advection scheme for the vertical turbulent diffusion parameterization. Both simulations, with and without wind farms, were continuously run over the period of 10 years from 2008—2017. As initial and lateral boundary conditions for the present experiments, data from continuous coastDat3 simulation started in 1979 and available hourly at a horizontal grid resolution of 0.11° (~ 11 km) are used^45^. The coastDat3 atmospheric simulation used the initial and boundary conditions from the European Centre for Medium-Range Weather Forecast (ECMWF) ERA-Interim reanalysis data available in 6 hourly intervals at a horizontal grid resolution of 0.703°^46^.

In this experiment, a scenario simulation was performed considering all areas with existing and planned OWFs in the North Sea according to the 2015 planning status^47^. The planning status of OWFs in the North Sea is rapidly changing every year; the latest changes in the German Wind Sea laws and other countries’ development plans will likely change the plans for future deployment of OWFs in the North Sea. Hence, this simulation is an exemplary illustration of the potential future effects of large-scale OWF deployment rather than a realistic prediction of the future situation. These are continuous simulations performed for a multiyear period from 2008–2017 to account for a range of different weather conditions in assessing the impact of large-scale OWF development on the sea surface fluxes of heat and momentum. Hereafter, “CCLM_WF” and “CCLM” refer to the COSMO-CLM simulation with wind farm parameterization and the control simulation without it, respectively. To quantify the impact of large OWFs in the North Sea, the change in climate variables is compared with the interannual variability and the climate change signals calculated using coastDat3 data (Tables 1 and 2) and the data from the North Sea Region Climate Change Assessment (NOSCCA) report^48^.

**Table 1:** Mean standard deviation per decade derived from regional reanalysis driven hindcasts (column 2—7), in comparison to the impact of wind farms (column 8).

	Means over wind farm areas			Mean over the North Sea basin			Means over wind farm areas
	cD3-NCEP 0.165° 1950–2020	cD3-ERA-Interim 0.11 1980–2018	CCLM 0.02° 2008–2017	cD3-NCEP 0.165° 1950–2020	cD3-ERA-Interim 0.11 1980–2018	CCLM 0.02° 2008–2017	Change CCLM_WF – CCLM over wind farms
PREC (mm day^−1^)	3.882	3.655	3.844	2.475	2.277	2.416	0.09
WS (ms^−1^)	3.624	3.598	3.967	2.174	2.155	2.409	− 0.52
2 m Temp (K)	4.623	4.471	4.693	2.766	2.651	2.781	0.09
SH (Wm^−2^)	25.803	25.489	22.493	16.926	16.073	14.470	− 1.86
LH (Wm^−2^)	42.818	50.989	43.329	25.383	29.256	25.215	− 0.40
LW (Wm^−2^)	29.478	28.585	28.676	17.952	17.415	17.492	− 0.95
SW (Wm^−2^)	89.469	89.891	85.623	53.785	53.505	51.449	− 1.19
NH (Wm^−2^)	124.958	129.719	113.413	75.309	75.9135	66.924	− 2.02
Cloud cover (1)	0.301	0.299	0.263	0.182	0.182	0.222	0.02
2 m sp hum (g kg^−1^)	2.079	2.15	2.18	1.221	1.255	1.28	− 0.09

**Table 2:** Trends per decade (1950–2018 and 1980–2018) derived from regional reanalysis driven hindcasts (column 2—4) , in comparison to the impact of wind farms (column 5) over the North Sea. SH, LH, LW and NH are defined positive upward, SW positive downward.

	cD3-NCEP 0.165° (1950–2018)	cD3-NCEP 0.165° (1980–2018)	cD3-ERA-Interim 0.11 (1980–2018)	Change CCLM_WF—CCLM over wind farms	Comments
PREC (mmday^−1^)	− 0.009	0.016	0.017	0.092	WF signal stronger than trend
WS (ms^−1^)	0.067	0.007	− 0.008	− 0.52	Trend model depended
2 m Temp (K)	0.179	0.217	0.195	0.09	WF 50% add on to climate change
SH (Wm^−2^)	0.189	0.199	− 0.035	− 1.86	Sign of trend model depended; WF signal stronger
LH (Wm^−2^)	1.949	1.486	0.880	− 0.40	WF signal compensates parts of trend
LW (Wm^−2^)	0.710	0.419	0.069	− 0.95	WF signal compensates parts of trend
SW (Wm^−2^)	1.391	0.665	− 0.176	− 1.19	Sign of trend model depended; WF signal stronger
NH (Wm^−2^)	1.457	1.439	1.462	− 2.02	WF signal overcompensates trend
Cloud cover (1)	− 0.008	− 0.005	− 0.003	0.02	WF signal overcompensates trend
2 m sp hum (g kg^−1^)	0.039	0.067	0.081	− 0.09	WF signal compensates trend

## Results and discussion

In this section, the impact of large OWFs on sea surface heat fluxes (radiative and turbulent fluxes), 10 m wind speed, near-surface air temperature, specific humidity, cloud cover, and precipitation in the North Sea were discussed in detail. The discussion focuses on annual and seasonal timescales for the dominating southwesterly winds (200–280°). Therefore, from the hourly dataset those time steps were selected of each grid point where the wind was blowing from directions between 200 and 280° to calculate the mean values. The annual and seasonal mean differences for all wind directions (0—360°) are shown in the Supplementary Information (SI). In the case of all wind directions, mean values from the hourly datasets were used. For completeness, the analysis of the model quality without wind farm effects is shown in SI for the mean annual cycles of the turbulent fluxes, radiative fluxes, 10 m wind speed, 2 m air temperature, 2 m specific humidity, cloud cover, and precipitation for the same period of 2008—2017 (Supplementary Note 1). The direct effect of the implemented wind farms on the wind speed and wind farm wakes has been validated against mast and in situ airborne measurements in a previous article^42^.

The net upward heat flux NH (Eq. 1) is defined as the sum of turbulent fluxes and radiative fluxes and is positive upwards^38^.

1 $$NH=LH+SH+LW-SW$$

The net upward latent heat flux (LH), net upward sensible heat flux (SH), and net surface upwelling longwave radiation (LW) are defined as positive upwards, while net surface downwelling shortwave radiation (SW) is positive downwards.

The total mean difference (MD: in absolute values, and, except for temperature, in percentages) and root mean square error (RMSE) of annual and seasonal mean values between the CCLM_WF and CCLM over the wind farm areas are used as statistical measures in the following discussion.

## Impact of OWFs on wind speed and TKE

**Figure 2:**
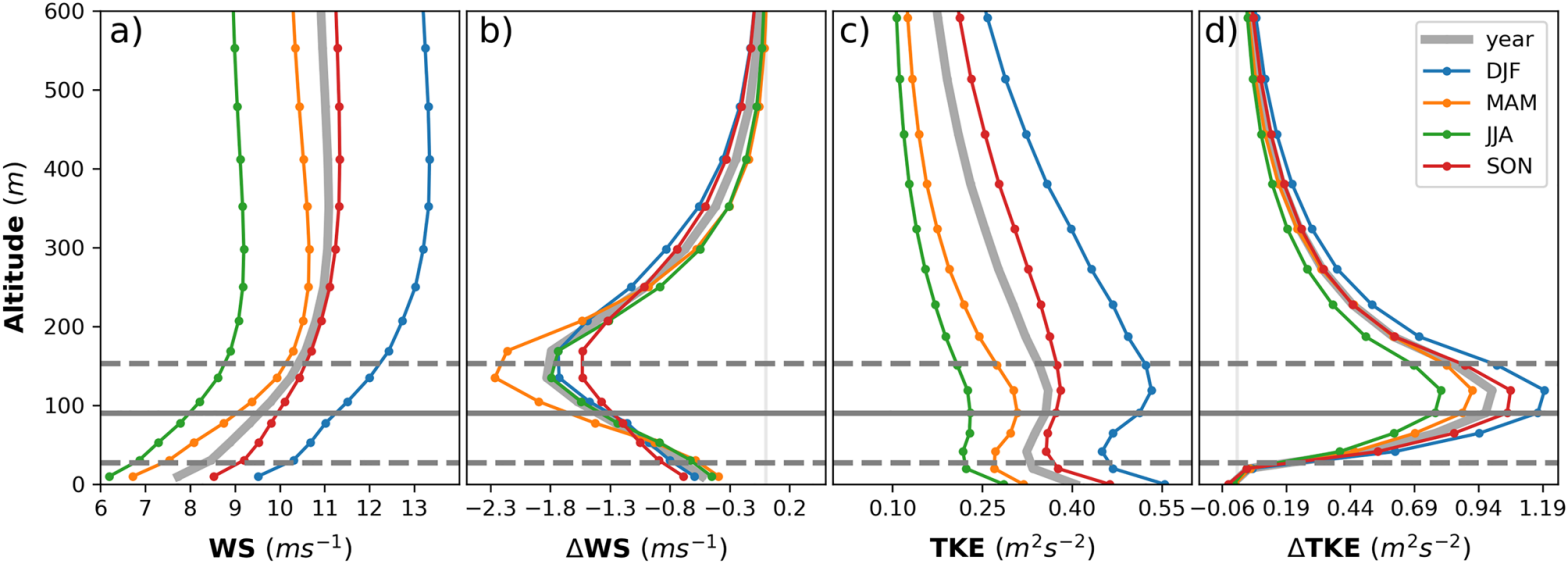
Mean vertical profiles of the CCLM for (**a**) wind speed and (**c**) turbulent kinetic energy and differences between the CCLM_WF and CCLM in vertical profiles of the (**b**) wind speed and (**d**) turbulent kinetic energy over the wind farm areas in 2008–2017 for all wind directions (0–360°). Solid circles indicate the model’s main levels (**a**) or half levels (**b**). The solid gray line indicates the hub height (90 m) of the turbine, whereas dotted gray lines indicate the lower (27 m) and upper (153 m) tips of the rotor.

The results show that large OWFs strongly alter the vertical atmospheric structure by reducing the wind speed and increasing the TKE, which increases vertical mixing mainly within and above the rotor area (Fig. 2). This enhanced vertical mixing changes the vertical profile of the temperature and specific humidity below, above and within the rotor area. On average, these changes in vertical atmospheric structure generated by the wind farms propagate approximately 600 m above the mean sea level for the turbine size (hub height 90 and rotor diameter 126 m) used in this study (i.e., approximately 450 m above the rotor area). Horizontally, wake effects associated with the offshore wind reach up to 35—40 km downwind of the wind farm^42^.

The conversion of KE into electric power by wind turbines reduces the wind speed and increases the TKE within the wind farm and in the downwind wakes. These changes in the wind speed and TKE are greatest in the atmospheric layers between the hub (90 m) and the tip height (153 m). Figure 2 shows the mean vertical profiles of the CCLM and the differences between the CCLM_WF and CCLM in wind speed and TKE over the wind farm areas. The changes in wind speed and TKE enhance the vertical mixing mainly within and above the rotor area that reaches approximately 450 m above turbine height. The maximum annual mean wind speed is approximately 1.8 ms^−1^ (18%) lower in the CCLM_WF than in the CCLM, which is consistent with a previous numerical study^49^ based on the same wind farm parametrization used in this study. An increase of approximately 0.9 m^2^s^−2^ (275%) is found in TKE over the wind farms in the CCLM_WF compared to that over the CCLM (Fig. 2). This increase in TKE is small compared to that reported by other studies using the WRF model^49,50^. The greater the TKE increase generated in WRF might be connected to a recently found bug in the implementation of the wind farm parameterization in WRF and the consequentially excessive value of the coefficient that relates turbine electro technical losses to TKE^51^.

The wind speed deficits at hub height due to offshore wind farm installations are higher during spring (2.3 ms^−1^ 22%) and summer (1.8 ms^−1^ 21%) than during winter (1.7 ms^−1^ 14%) and autumn (1.5 ms^−1^ 15%), even though the mean wind speed is higher in the North Sea during these seasons (Fig. 2b). Higher wind speed deficits during spring and summer could be due to atmospheric conditions, which are generally stable in the North Sea^42^. Weak atmospheric mixing during stable atmospheric conditions leads to higher and longer wake effects^42^. However, the wind speed deficits at the lowest atmospheric level are slightly higher during winter and autumn than during the other seasons.

The maximum increase in TKE is approximately 1.2 m^2^s^−2^ (186%) and 1.1 m^2^s^−2^ (260%) during winter and autumn, respectively, in the CCLM_WF compared to the CCLM over the wind farms (Fig. 2d). This change in the TKE in the CCLM_WF is slightly smaller during spring (0.9 m^2^s^−2^) and summer (0.79 m^2^s^−2^) compared to other seasons over the wind farm areas. At the lowest atmospheric level, there is a slight decrease in the TKE of CCLM_WF compared to that of CCLM for all seasons.

**Figure 3:**
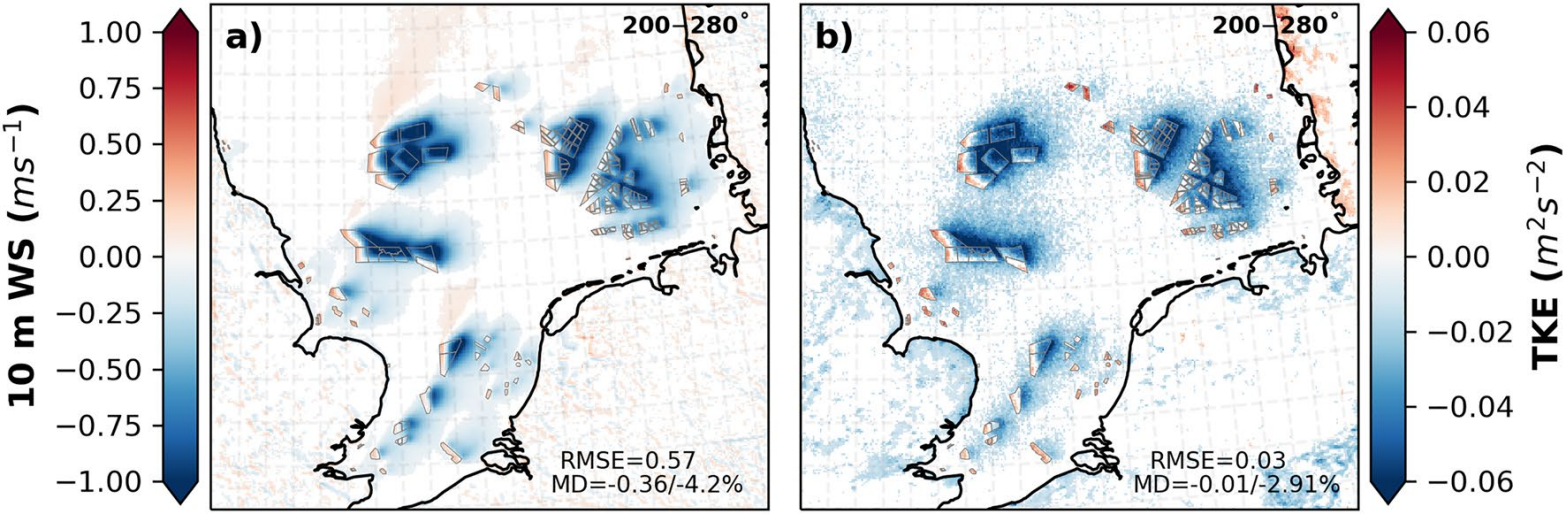
Annual mean difference between the CCLM_WF and CCLM in (**a**) 10 m wind speed and (**b**) surface turbulent kinetic energy outside and inside the wind farms for the prevailing wind directions of 200–280° in 2008–2017. Root mean square errors (RMSE) and mean differences (MD) over the wind farm areas in 2008–2017 are given in the legend. This figure was created with Matplotlib (Hunter, J. D., Matplotlib: a 2D graphics environment. Computing in Science and Engineering 9, 2007) and Cartopy (Met office, Cartopy: a cartographic python library with a matplotlib interface. Exeter, Devon, https://scitools.org.uk/cartopy, 2015).

As seen in the vertical profiles, the behavior of wind speed and TKE at the lowest layer is different from the layers within the rotor area and at hub height. Figure 3 shows the differences between the CCLM_WF and CCLM at a 10 m wind speed and TKE at the lowest atmospheric level for southwesterly winds (200–280°). The deficits in the 10 m wind speed reached up to 1 ms^−1^ at the wind farm areas in the CCLM_WF. The reduction effect was weaker than at hub height, but the spatial extent is similar. However, wind speed acceleration at 10 m height (up to 0.5 ms^−1^) is found in small wind farms and at the upstream edge of the wind farms (Fig. 3a). The annual mean values of the 10 m wind speed and TKE at the lowest atmospheric level are shown in the SI (Fig. SI 2). This acceleration in the near-surface wind is more pronounced in spring and summer when the atmosphere is comparatively more stable in the North Sea than in other seasons (Fig. SI 2 and SI 3). Such below rotor wind acceleration was also observed in wind farm measurements^52^ and model simulations^53,54^. The annual mean 10 m wind speed deficits in the CCLM_WF compared to those in the CCLM are approximately 0.38 ms^−1^ (4%) in the wind farm areas, with similar differences in summer (MD = − 0.38 ms^−1^/5%) and slightly higher values in autumn (MD = − 0.50 ms^−1^/5%) compared to other seasons. Due to the increase in wind speed at the upstream edges of the wind farms during spring and summer, the area mean difference between the CCLM_WF and CCLM for wind speed at 10 m height was smaller during these seasons (Fig. 2 and Fig. SI 3).

The wind turbine increases TKE through the vertical column. However, at the lowest atmospheric level, a slight decrease (up to 0.05 m^2^s^−2^) is found in the mean values of CCLM_WF compared to those of CCLM (Fig. 2b). The decrease in the wind shear near the surface due to the wind speed deficit results in TKE reduction^29^. Due to near-surface wind acceleration, TKE also increases at the upstream edge of the wind farms. While the annual mean values of surface TKE are reduced by approximately 2% over the wind farm area, the reduction in autumn is stronger, with approximately 0.03 m^2^s^−2^ (5%) in the CCLM_WF than in the CCLM (Fig. SI 3).

## Impacts of OWFs on temperature and specific humidity

**Figure 4:**
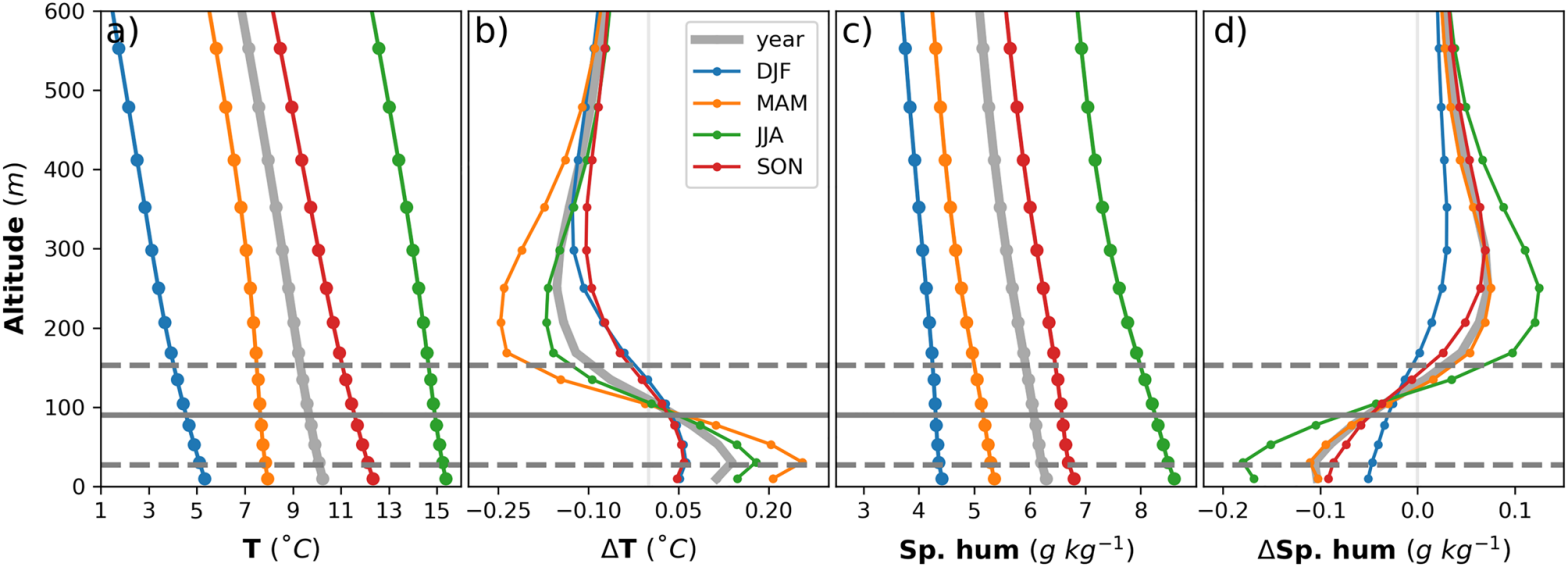
Mean vertical profiles of the CCLM for (**a**) temperature and (**c**) specific humidity and differences between the CCLM_WF and CCLM in vertical profiles of the (**b**) temperature and (**d**) specific humidity over the wind farm areas in 2008–2017. Solid circles indicate the model’s main levels (**a**) or half levels (**b**). The solid gray line indicates the hub height (90 m) of the turbine, whereas dotted gray lines indicate the lower (27 m) and upper (153 m) tips of the rotor.

A wind turbine mixes the air column by carrying moist air aloft^29^. Figure 4 shows the mean vertical profiles of the CCLM and the differences between the CCLM_WF and CCLM for temperature and specific humidity over the wind farm areas. Above the hub height, the maximum annual mean decreases in temperature of approximately 0.15 °C and increases in specific humidity of approximately 0.07 g kg^−1^ (1.3%) were found in the CCLM_WF compared to the CCLM over the wind farms. These differences are more prominent in spring, with temperatures higher by − 0.24 °C, and in summer, with specific humidity higher by 0.12 g kg^−1^ (1.6%) than those in other seasons (Fig. 4b and d). The wind turbines changed the vertical profile of the atmosphere mainly within the wind farm area due to enhanced vertical mixing: atmospheric levels below the hub height become drier and warmer, with the highest differences at the edge of the lowest rotor tip, which is 27 m above the sea surface (Fig. 4). Below the hub height, the annual mean values of temperature increased by a maximum of approximately 0.12 °C (Fig. 4b), and specific humidity decreased the most by approximately 0.1 g kg^−1^ (2%) in the CCLM_WF compared to those in the CCLM over the wind farm areas (Fig. 4d). On a seasonal timescale, the increase in temperature was highest during spring (maximum 0.25 °C), and the decrease in specific humidity was strongest in summer (maximum 0.18 g kg^−1^; 2.1%). The change in the entire vertical profile of the specific humidity and temperature in the CCLM_WF compared to that in the CCLM was more pronounced during spring and summer than during other seasons.

**Figure 5:**
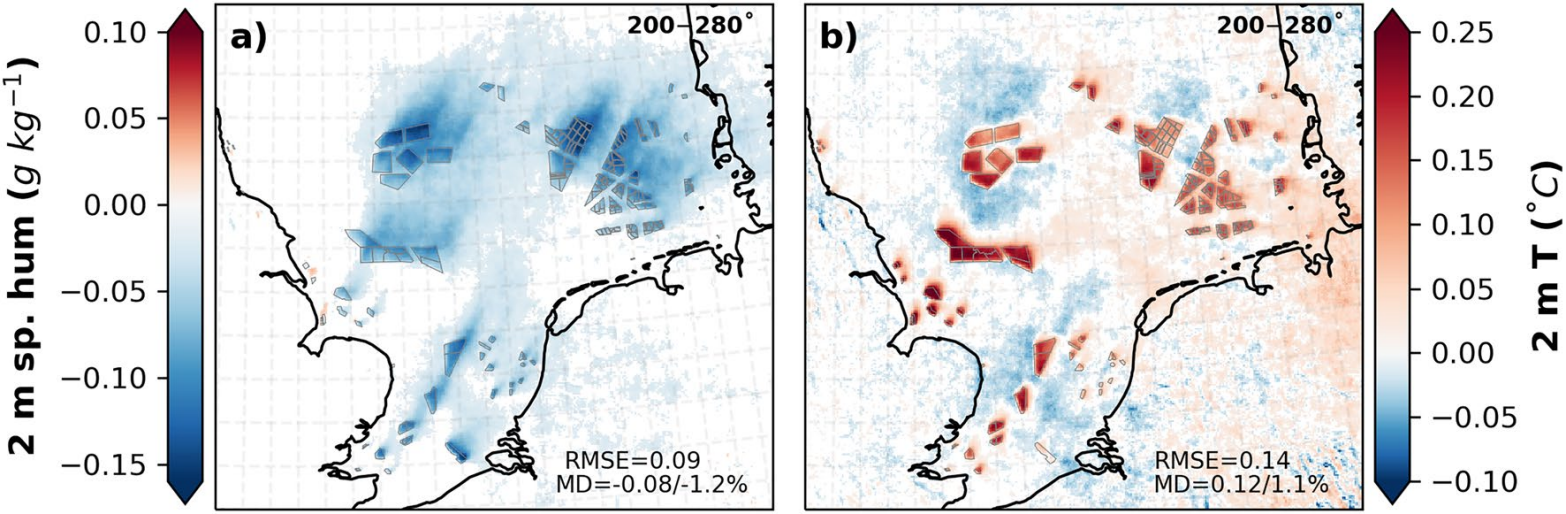
Annual mean difference between the CCLM_WF and CCLM in 2 m (**a**) specific humidity and (**b**) temperature outside and inside the wind farms for the prevailing wind directions of 200–280° in 2008–2017. Root mean square errors (RMSE) and mean differences (MD) over the wind farm areas in 2008–2017 are given in the legend. This figure was created with Matplotlib (Hunter, J. D., Matplotlib: a 2D graphics environment. Computing in Science and Engineering 9, 2007) and Cartopy (Met office, Cartopy: a cartographic python library with a matplotlib interface. Exeter, Devon, https://scitools.org.uk/cartopy, 2015).

The annual mean differences in 2 m specific humidity show that a reduction of up to 0.09 g kg^−1^ (1.3%) in the CCLM_WF compared to that in the CCLM was found in the wind farm areas and wakes (Fig. 5a), which was more pronounced in summer, with mean differences of 0.14 g kg^−1^ (Fig. SI 3). This decrease in moisture due to enhanced vertical mixing increases the 2 m temperature (Fig. 5b). The 2 m annual mean temperatures show an increase of up to 0.25 °C, mainly in the wind farm areas, in the CCLM_WF compared to that in the CCLM. On a seasonal timescale, the increase in the 2 m temperature was higher in spring (0.18 °C) and summer (0.11 °C) than in the other seasons (Fig. SI 3). Unlike the 2 m temperature, the reduction in 2 m specific humidity was more pronounced during summer and spring at 1.5%. A slight increase in the mean values of up to 0.05 °C was found in wake areas.

## Impact of OWFs on surface net heat flux

The results of this study show that the presence of wind farms reduces the 10 m wind speed mainly inside and outside downwind of the wind farms in the wake. In contrast, a slight increase in the 10 m wind speed was found at the upwind edge of the wind farms due to the wind channeling effect, which was more pronounced during spring and summer. In contrast to hub height, where the TKE was largely increased due to the wind turbine effect, TKE was found to decrease at the lowest atmospheric level in the areas where the 10 m wind speed reduced. TKE was also found to increase in areas where the 10 m wind speed increased. This change in the wind speed and TKE modified the turbulent fluxes. The spatial and temporal patterns of change in the LH flux were highly correlated with the change in 10 m wind speed and TKE. However, the SH flux was reduced in all seasons and areas of the wind farms. This shows that the change in the SH flux was mainly dominated by the temperature gradient between the sea surface and the lowest atmospheric layer together with wind speed.

**Figure 6:**
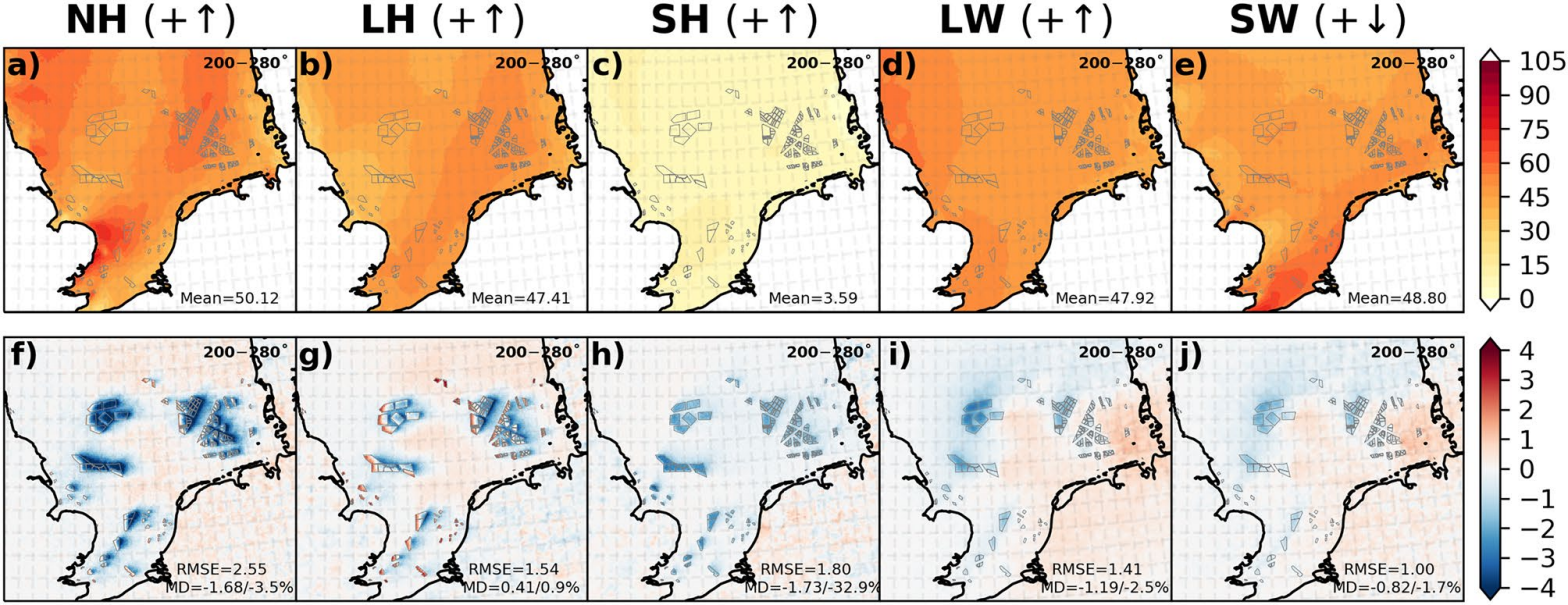
Annual mean values of the CCLM and mean differences between the CCLM_WF and CCLM for (**a**, **f**) net heat (NH) flux, (**b**, **g**) latent heat (LH) flux, (**c**, **h**) sensible heat (SH) flux, (**d**, **i**) net upwelling longwave (LW), and (**e**, **j**) net shortwave downwelling (SW) radiations outside and inside the wind farms for the prevailing wind directions of 200–280° in 2008–2017. Root mean square errors (RMSE) and mean differences (MD) over the wind farm areas in 2008–2017 are given in the legend. This figure was created with Matplotlib (Hunter, J. D., Matplotlib: a 2D graphics environment. Computing in Science and Engineering 9, 2007) and Cartopy (Met office, Cartopy: a cartographic python library with a matplotlib interface. Exeter, Devon, https://scitools.org.uk/cartopy, 2015).

The impact of wind turbines below the rotor area on wind speed and TKE modified the NH flux by influencing the turbulent (LH and SH) and radiative (LW and SW) fluxes. Figure 6 shows the mean values of the CCLM for the NH flux and its components and the differences between the CCLM_WF and CCLM over the wind farm area for the southwesterly winds (200—280°). The seasonal mean values of the NH flux and its components are shown in the SI (Fig. SI 4a). For these wind directions, the mean values of the NH flux ranged between 30 and 60 Wm^−2^ (Fig. 6a). The values were reduced in the CCLM_WF by up to 5% (MD = − 2.35 Wm^−2^) over the wind farm areas. The seasonal mean differences between the CCLM_WF and CCLM were approximately − 3.4 Wm^−2^ (− 4%) in winter and − 3.8 Wm^−2^ (− 3.5%) in autumn (Fig. SI 4a and Fig. SI 5).

## Impact of OWFs on turbulent fluxes

The differences in turbulent and radiative fluxes indicate that all the components of the NH flux were influenced by the wind farms due to the changes in the wind speed and TKE (Fig. 6). The impacts of wind farms on turbulent fluxes are of particular concern for the ocean, as these fluxes are the primary mechanism by which the ocean transfers heat to the atmosphere.

The annual mean values of the LH flux range between 30 and 50 Wm^−2^ for the southwesterly winds in the North Sea (Fig. 6b). The mean differences between the CCLM_WF and CCLM in the LH flux show an increase in values in the CCLM_WF by approximately 0.9% (MD = 0.4 Wm^−2^) for the southwesterly winds for the wind farm areas (Fig. 6g). These differences vary locally from 4 Wm^−2^ to − 4 Wm^−2^. The seasonal mean differences between the CCLM_WF and CCLM in the LH flux show decreased values in the CCLM_WF for autumn (MD = − 1.4 Wm^−2^/− 1.8%; Fig. SI 5) and winter (MD = − 0.2 Wm^−2^/− 0.3%). However, the mean values of the LH flux were found to increase during spring (MD = 1.8 Wm^−2^/9%) and summer (MD = 1.7 Wm^−2^/4%) in the CCLM_WF in comparison with those in the CCLM (Fig. SI 5). Similar increasing and decreasing patterns in LH fluxes were found for onshore wind farms at smaller magnitudes^29^.

The annual mean values of the SH flux ranged between 0 and 4 Wm^−2^ for the southwesterly winds in the North Sea (Fig. 6c). The sensible heat flux was generally reduced for all wind farm areas during all seasons (Fig. 6h, Fig. SI 5). The annual mean differences in the SH flux were reduced up to 2.5 Wm^−2^ (MD = − 1.9 Wm^−2^/− 28%) in the CCLM_WF compared to those of the CCLM. Seasonally, this reduction in the SH flux was most pronounced during winter (MD = − 1.9 Wm^−2^/− 33%) and spring (MD = − 2.4 Wm^−2^/− 44%) at the wind farms (Fig. SI 5. However, the relative differences were highest during the spring (MD = − 2.4 Wm^−2^/− 44%) due to the small summer mean value (− 5.6 Wm^−2^) compared to that of other seasons. The reduction in the negative SH fluxes in spring and summer refers to an increase in the fluxes from the atmosphere to the ocean. The SH flux was overall reduced within the wind farms and in wakes, whereas LH increased over the upstream edges of the wind farms and reduced over the downstream edges of the wind farms and in wakes. Some changes were found, mainly increases, over land in the eastern part of the North Sea coast.

Several factors can modify the turbulent fluxes, as shown in the bulk parameterizations (Eqs. 2 and 3):

2 $$LH= \rho {C}_{H}{L}_{v}WS \left({q}_{s}-{q}_{a}\right)$$

3 $$SH= \rho {C}_{\theta }{C}_{pd}WS\left({T}_{s}-{T}_{a}\right)$$

where *ρ* is the air density, *WS* is the 10 m wind speed, *L*_*v*_ is the specific latent heat of evaporation depending on the SST, and *q*_*s*_ (*T*_*s*_) and *q*_*a*_ (*T*_*a*_) are the specific humidity at saturation/temperature at the surface and lowest atmospheric layer, respectively. Furthermore, *C*_*pd*_ is the heat capacity of the air, and *C*_*H*_ and *C*_*θ*_ are the transfer coefficients for turbulent moisture transfer and turbulent heat exchange, respectively. The transfer coefficients *C*_*H*_ and *C*_*θ*_ are defined in the COSMO-CLM as follows^44,55^:

4 $${C}_{H}={C}_{\theta }= \frac{{K}_{H}^{ke1}}{WS {r}_{h}}$$

where ${K}_{H}^{ke1}$ is the turbulent diffusion coefficient for heat (at *ke1,* the surface), and *r*_*h*_ is the total resistance of the lowest model layer.

The surface turbulent fluxes mainly change due to changes in the specific humidity/temperature contrast between the surface and the lowest atmospheric layer and indirectly due to variations in the wind speed (Eqs. 2 and 3). Additionally, the reduction in TKE in the lowest atmospheric layer leads to a decrease in ${K}_{H}^{ke1}$ (Eq. 4). This means that reduced mixing in the lowest model layer leads to a reduction in the turbulent fluxes. The results show that changes in the LH flux are strongly influenced by the changes in the 10 m wind speed and TKE (Figs. 3, 6). Previous studies based on the same wind farm parameterization used in this study also reported a decrease in the LH heat flux when the lower atmospheric layer is warmer and drier with respect to the surface layer for onshore wind farms^29,56^. In contrast, a lower atmospheric layer that is moister and colder than the surface layer leads to an increase in LH heat fluxes^27^.

**Figure 7:**
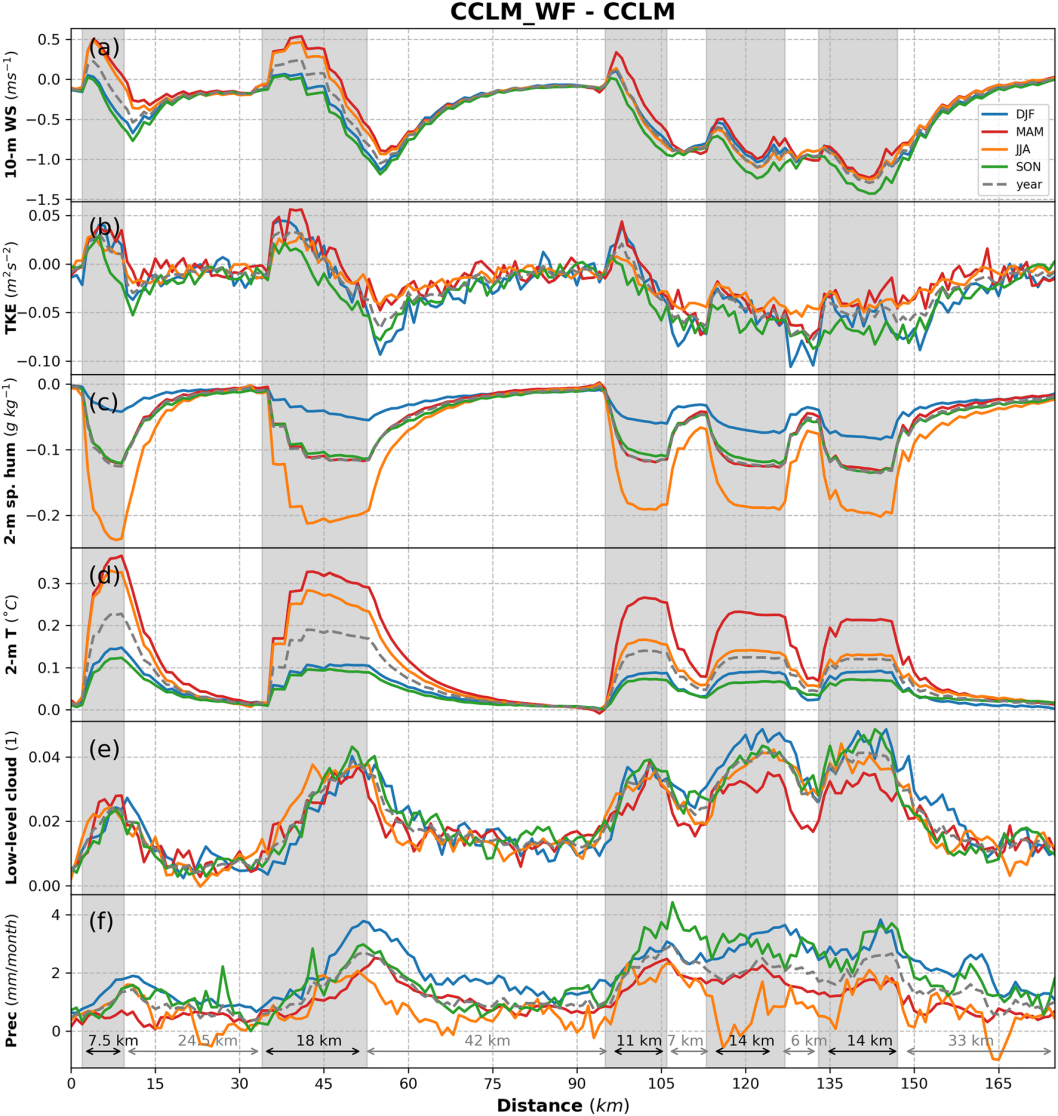
Transects of the seasonal (colored, see legend) and yearly deviation from means (dashed gray) for (**a**) net heat flux, (**b**) latent heat, (**c**) sensible heat, (**d**) net longwave upwelling radiation, and (**e**) net shortwave downwelling radiation for the prevailing wind directions of 200–280° in 2008–2017 taken at transect 1 (Dogger Bank, Fig. 1) latitude 54.4°N–55.8°N and longitude 0.8°E–3.15°E. Gray sectors indicate the wind farm positions.

**Figure 8:**
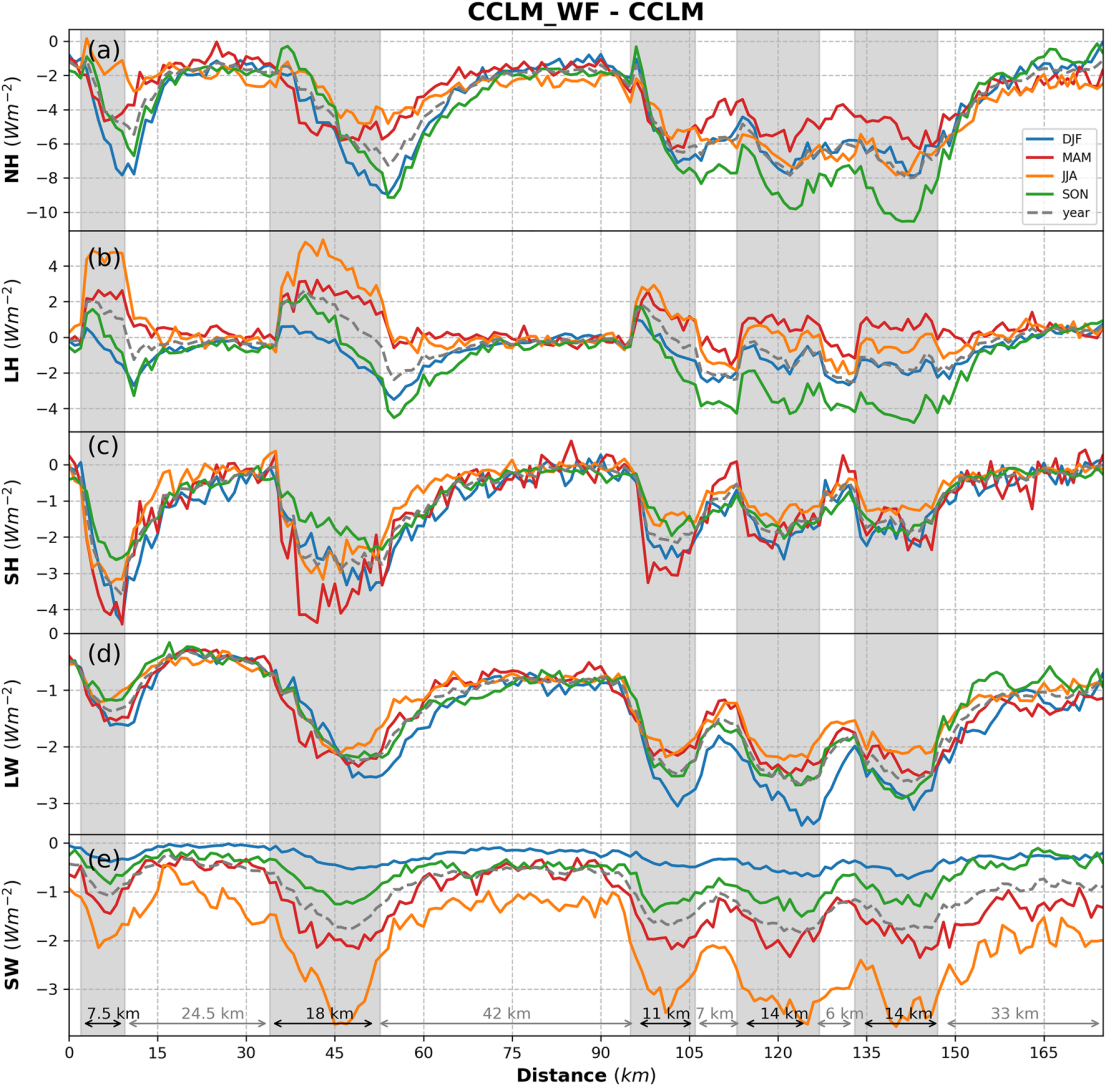
Transects of the seasonal (colored, see legend) and yearly deviation from means (dashed gray) for (**a**) 10-m wind speed, (**b**) turbulent kinetic energy, (**c**) 2-m specific humidity, (**d**) 2-m temperature, (**e**) low cloud amount, and (**f**) total precipitation for the prevailing wind directions of 200–280° in 2008–2017 taken at transect 1 (Dogger Bank, Fig. 1) latitude 54.4°N–55.8°N and longitude 0.8°E–3.15°E. Gray sectors indicate the wind farm positions.

Figures 7 and 8 show the transects along Dogger’s Bank (shown as line 1 in Fig. 1) of the NH flux together with its components (Fig. 7) and atmospheric variables potentially affected by the action of wind turbines and, in turn, changing the turbulent and radiative fluxes (Fig. 8). The transects of the LH flux (Fig. 7b) show similar increasing and decreasing patterns as those found in the 10 m wind speed and TKE (Fig. 8a, b): they show a slight increase in the 10 m wind speed at the upstream edges and a decrease in the TKE downstream of the wind farms, illustrating and confirming the former findings. The mean wake effects at a height of 10 m reach more than 25 km downwind. Seasonally, increases in the LH flux are more prominent in the spring and summer when the near-surface wind acceleration is stronger compared to other seasons (Figs. 7, 8). The differences in 2 m specific humidity show that the atmosphere near the surface is drier, especially during summer, in the CCLM_WF than in the CCLM (Fig. 8c). In the case of the sensible heat flux (Fig. 7c), changes are primarily influenced by changes in the temperature contrast between the surface and lowest atmospheric layer and wind speed at the lowest atmospheric model layer, as the sea surface temperature in both the CCLM_WF and CCLM simulations is prescribed by the ERA-Interim forcing data. In winter, when the sea is warmer than the atmosphere, the heat transfer to the atmosphere is reduced due to the reduction in the temperature gradient. In spring, a reduction was not found in the temperature gradient, but the temperature difference was more strongly negative, which again leads to a reduction in sensible heat flux. This reduction means more heat transfer from the atmosphere to the ocean in the spring.

In contrast to our results, a study using the WRF regional climate model reported an increase in turbulent fluxes within the wind farm and in the wake area^12^. The increase in turbulent fluxes in the WRF model simulation may have been due to the higher TKE, which is due to the excessive value of the TKE coefficient in the model^51^. Such an increase in surface heat fluxes was also reported in a study based on LES^34^. However, a numerical study performed with the mesoscale model METRAS over the German Bight region also found a reduction in turbulent fluxes^32^. Another study using the global Community atmospheric model (CAM5) found a change in turbulent fluxes within the range of ± 1 Wm^−2^, with an overall increase in the mean LH flux of approximately + 0.16 Wm^−2^ and a decrease in the mean SH flux of approximately 0.5%^29^. Moreover, the experimental evidence based on wind-tunnel experiments confirms the reduction in surface heat fluxes (approximately 4%) in the case of staggered wind farms^23^.

## Impact of OWFs on radiative fluxes

Wind farms also impact the radiative flux (LW and SW) by modifying low clouds. The annual mean values of the LW radiation range between 35 and 70 Wm^−2^ for the southwesterly winds in the North Sea (Fig. 6d, Fig. SI 4d). The annual mean values of the net surface upwelling longwave radiation LW are found to be reduced by up to 2 Wm^−2^ (MD = − 1.2 Wm^−2^/− 2.5%) in the CCLM_WF compared to those in the CCLM over the wind farm areas (Fig. 6i). In the wake areas, the mean reduction of approximately 0.5 Wm^−2^ in LW was found downwind of the wind farms. On a seasonal timescale, differences between the CCLM_WF and CCLM in net surface upwelling LW radiation were found to vary within a small range (− 2.1% to − 2.9%). The pattern of LW changes outside the wind farms in connection to low cloud cover is discussed later.

The annual mean values of the SW flux ranged between 20 and 70 Wm^−2^ for the southwesterly winds in the North Sea (Fig. 6e, Fig. SI 4e). Similar to the net surface upwelling LW radiation, the net surface downwelling radiation SW was found to decrease in the CCLM_WF compared to that in the CCLM by up to 2.0 Wm^−2^ (MD = − 0.8 Wm^−2^/− 1.7%) over the wind farm areas. An increase of up to 1.5 Wm^−2^ was found in the wake areas east of the wind farms (Fig. 6j). The change in the SW due to wind farms was higher during spring and summer (Fig. SI 5).

## Impact of OWFs on cloud cover and precipitation

**Figure 9:**
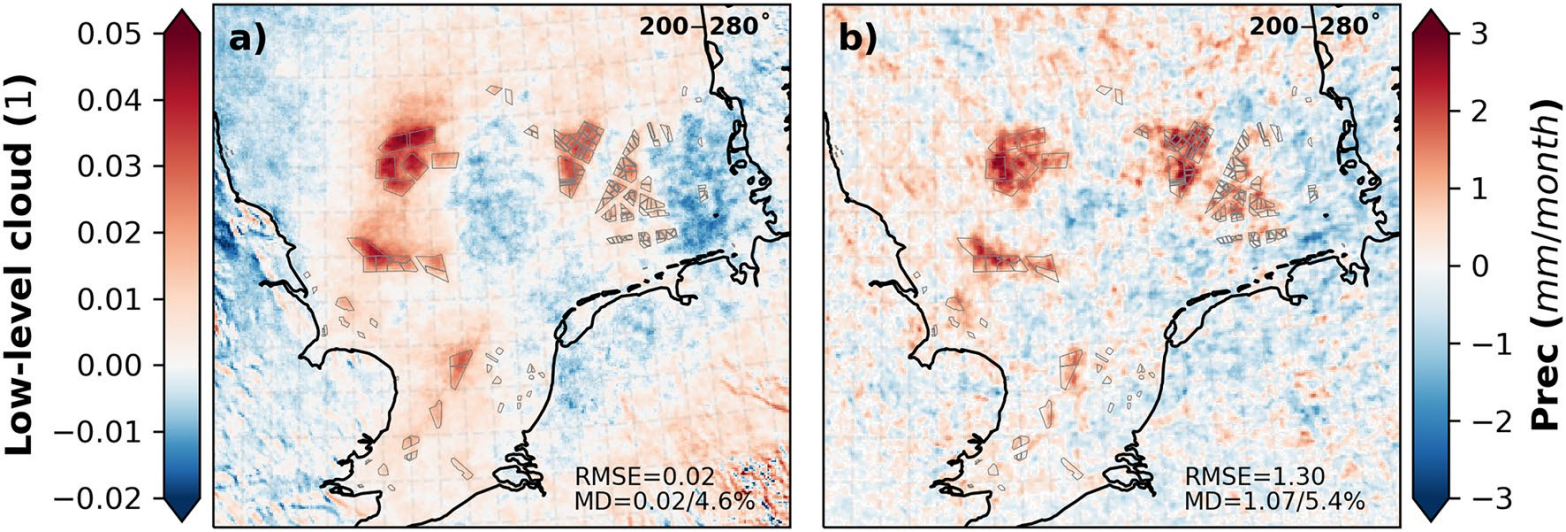
Annual mean difference between the CCLM_WF and CCLM for (**a**) low cloud amount and (**b**) total precipitation outside and inside the wind farms for the prevailing wind directions of 200–280° in 2008–2017. Root mean square errors (RMSE) and mean differences (MD) over the wind farm areas in 2008–2017 are given in the legend. This figure was created with Matplotlib (Hunter, J. D., Matplotlib: a 2D graphics environment. Computing in Science and Engineering 9, 2007) and Cartopy (Met office, Cartopy: a cartographic python library with a matplotlib interface. Exeter, Devon, https://scitools.org.uk/cartopy, 2015).

The change in the radiative fluxes was primarily influenced by the change in low clouds, which is defined as the fraction of cloud area at levels below 700 hPa. The coverage with low clouds increased over the wind farm area (Fig. 8e and 9a). The annual mean values showed an increase of up to 0.05 (MD = 0.02/4.3%) in low clouds in the CCLM_WF compared to in the CCLM over the wind farm areas. Seasonally, a slight variation in low clouds, ranging from4% to 5% (Fig. 8e, Fig. SI 3), was found in the CCLM_WF compared to the CCLM. Low clouds increased due to flow convergence and uplift at the upstream edge of wind farms^29^. The uplift by wind turbines increases the moisture aloft, which then increases the relative humidity and cloud fraction due to adiabatic cooling. A slight reduction in low clouds was found downstream of the wind at greater distances to the wind farms (Fig. 9a, Fig. SI 3). This decrease in low clouds occurs due to flow divergence in the wakes and diabatic warming^29^. The transect along Dogger Bank shows that radiative fluxes follow the inverted patterns of low clouds (Figs. 7d, e, and 8e): The low clouds increase at the upstream edge of the wind farms and reach a maximum at the downstream edge (Fig. 8e). The change in the cloud patterns, which increase over the wind farms and a reduction downwind of the wind farms, has also been previously reported^32^. Similarly, the reduction in the surface net radiative fluxes reaches the maximum near the downstream edge of the wind farms.

It is important to note that an increase in the low clouds over the wind farm increases the surface downward LW radiation in the CCLM_WF compared to that in the CCLM (Fig. SI 6). As a result, the net surface upwelling LW radiation is reduced in the CCLM_WF. The additional shadowing effect of an increase in low cloud fractions reduces the net surface downward SW radiation.

The increase in low clouds lead to an increase in total precipitation over the wind farm areas in the CCLM_WF compared to the CCLM (Figs. 8f and 9b). The annual mean values showed an increase of approximately 1 mm/month (7%) in the total precipitation amount in the CCLM_WF over the wind farms. A slight decrease in precipitation (approximately 1 mm/month) was also found east of the wind farms in the CCLM_WF due to the decrease in low clouds in those areas (Fig. 9a), where increases in SW (Fig. 6j) were also found. On a seasonal timescale, the increase in precipitation in the CCLM_WF in comparison with that of the CCLM was slightly higher during winter and autumn, with approximately 1.3 mm/month (6.1 and 6.4%) over the wind farm area compared to that of other seasons (Fig. SI 3f.). As the low cloud changes mainly reach their maximum at the downstream edge of the wind farms, the precipitation changes were mostly found to reach their maximum at the downstream edge (Fig. 8f). The transect along the German Bight (shown as line 2 in Fig. 1) is shown in SI (Fig. SI 7 and Fig. SI 8).

## Impact of OWFS on the net heat flux and its components in all wind directions (0—360°)

**Figure 10:**
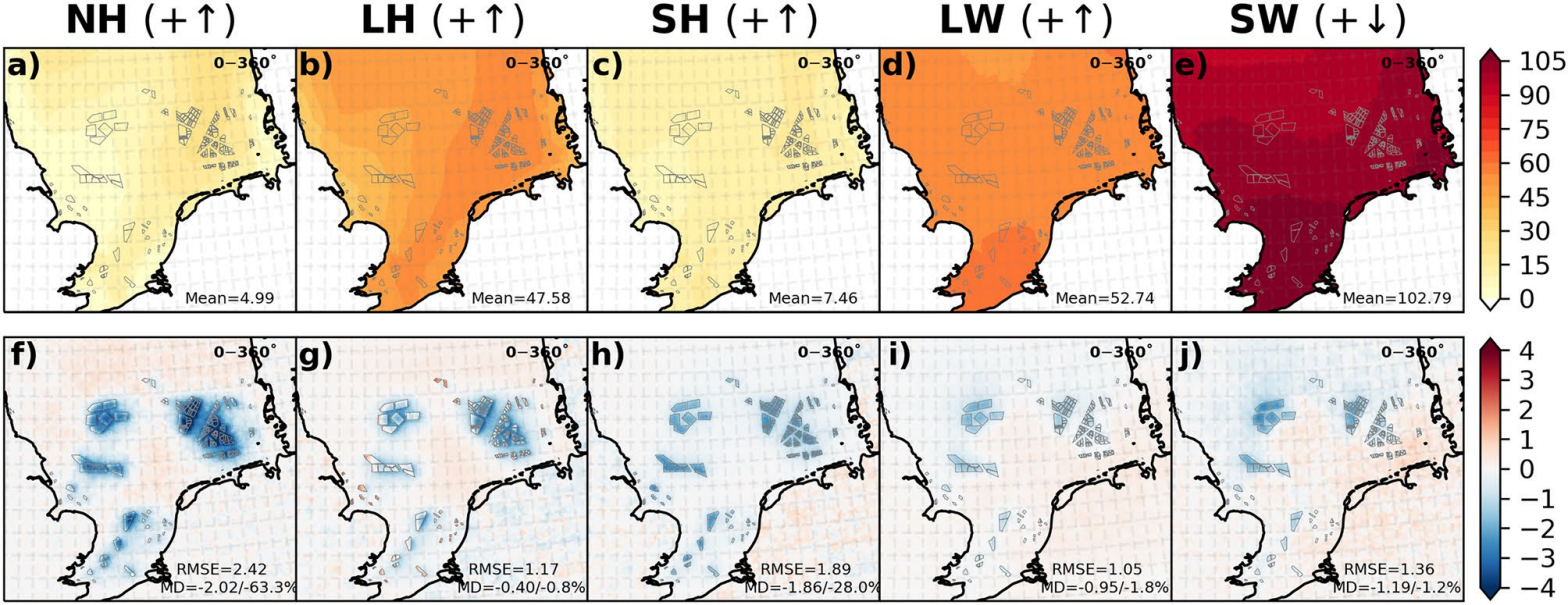
Annual mean values of the CCLM and differences between the CCLM_WF and CCLM for (**a**, **f**) net heat (NH) flux, (**b**, **g**) latent heat (LH) flux, (**c**, **h**) sensible heat (SH) flux, (**d**, **i**) net upwelling longwave (LW), and (**e**, **j**) net shortwave downwelling (SW) radiations outside and inside the wind farms for the prevailing wind directions of 0–360° in 2008–2017. Root mean square errors (RMSE) and mean differences (MD) over the wind farm areas in 2008–2017 are given in the legend. This figure was created with Matplotlib (Hunter, J. D., Matplotlib: a 2D graphics environment. Computing in Science and Engineering 9, 2007) and Cartopy (Met office, Cartopy: a cartographic python library with a matplotlib interface. Exeter, Devon, https://scitools.org.uk/cartopy, 2015).

In the previous sections, the impact of OWFs on the different climate variables for the prevailing southwesterly winds (200—280°) was discussed in detail. This constraint was used to show the possible variability of the spatial extent of the influences. To address the overall impact of the wind farms, the annual and seasonal mean values and differences between the CCLM_WF and CCLM for all wind directions (i.e., 0—360°) are briefly presented here for the surface downward NH flux and its components (Fig. 10, Fig. SI 9 and SI 10). The annual mean values showed a strong reduction (MD = − 2.0 Wm^−2^/− 63%) in the surface upwelling NH flux in the CCLM_WF compared to those in the CCLM (Fig. 10f), and the reductions were strongest during winter and autumn (Fig. SI 10a). The relative difference in the NH flux between the CCLM_WF and CCLM was high because of the low annual mean value of NH in the CCLM (5 Wm^−2^, Fig. 10a). The annual mean value of the LH flux in the North Sea was approximately 47.6 Wm^−2^ (Fig. 10b). It was slightly reduced (− 0.4 Wm^−2^/0.8%) in the CCLM_WF in comparison with those of the CCLM at the wind farm areas due to smoothing of the near-surface wind acceleration in the mean wind speed (for wind directions 0—360°) at the upstream edges of the wind farms (Fig. 10 g, Fig. SI 7). In the case of southwesterly winds (i.e., 200—280°), the near-surface wind acceleration was more pronounced, increasing the TKE and LH fluxes compared to those of all wind directions (i.e., 0—360°). On a seasonal timescale, the LH flux increased during spring (MD = 1.1 Wm^−2^/5%) and summer (MD = 1.4 Wm^−2^/4%) and decreased in winter (MD = − 1.7 Wm^−2^/3%) and autumn (MD = − 2.5 Wm^−2^/− 3%) in the CCLM_WF compared to the fluxes in the CCLM (Fig. SI 10b). The annual mean value of the SH flux in the North Sea was approximately 7.5 Wm^−2^ (Fig. 10c). Similar to the means over southwesterly wind cases, the SH flux was reduced in all seasons (highest relative difference (− 806%) found in summer), with annual mean differences up to − 1.9 Wm^−2^ (28%) over the wind farm areas (Fig. SI 10). Again, the reduction in the negative SH fluxes in spring and summer referred to an increase in the fluxes from the atmosphere to the ocean. The annual mean value of the net upwelling LW radiation in the North Sea is approximately 52.7 Wm^−2^ (Fig. 10d). The net upwelling LW radiation was also reduced over the wind farm area, with an annual mean difference of − 1 Wm^−2^ (− 2%) in the CCLM_WF. The reduction in the net LW radiation was higher during winter (− 1.2 Wm^−2^/− 2%) than during the other seasons (Fig. SI 10). A slight increase in the net LW radiation east of the wind farms was also found here, which was more prominent in the case of southwesterly winds (Figs. SI 5 and SI 10). The annual mean differences showed that the net SW radiation was reduced by approximately − 1.2 Wm^−2^ (− 1.2%) in the CCLM_WF compared with that of the CCLM. The mean differences were higher during summer (− 1.9 Wm^−2^/1%), whereas the relative differences were higher during winter (MD = − 0.5 Wm^−2^/− 2%). For completeness, the annual and seasonal mean values and differences for 10 m wind speed, turbulent kinetic energy, 2 m specific humidity, 2 m temperature, low cloud amount, and precipitation outside and inside the wind farms for all wind directions (0—360°) in 2008–2017 are shown in SI (Fig. SI 11 and SI 12).

## Discussion and conclusion

The results show that large OWFs alter the vertical atmospheric structure in the wind farm areas and in their vicinity by reducing the wind speed and increasing the TKE, which increases vertical mixing. The enhanced vertical mixing induced by the wind farms changes the vertical profile of the temperature and the specific humidity in and over the wind farms. Such effects are more pronounced during spring and summer than in other seasons. Vertically, the disturbance produced by the OWF turbines reaches approximately 600 m above mean sea level (i.e., approximately 450 m above the rotor area) when using the chosen turbines with a hub height of 90 m and rotor diameter of 63 m. This also confirms that the energy extracted by wind turbines is replenished mainly by large-scale pressure forces within the boundary layer, as shown in a recent theoretical study^8^. Horizontally, the mean wake effects generated by wind farms at the hub height extend, on average, up to 35—40 km beyond the farm areas^42^.

The results show that the impact of OWFs on sea surface fluxes is seasonally variable. The impacts of wakes generated by the large OWFs and near-surface wind acceleration were stronger for prevailing southwesterly winds (200—280°) than for the overall mean winds (0—360°).

The reduction in annual mean values of NH flux indicates that the heating of the atmosphere from the sea surface over OWFs and wake areas was reduced compared to boundary-layer flow without wind farms. The impact of OWFs on surface fluxes is spatially localized and overall smaller than the interannual variability of heat fluxes. However, it is comparable in magnitude to present-day climate change impacts (Table 1). On average, the NH flux was found to be reduced by approximately 63% over the wind farm areas in the CCLM_WF compared to that of the CCLM when averaged for all wind directions (0—360°). Generally, in winter and autumn, net heat transfer takes place from the ocean to the atmosphere, while it is reversed in summer and spring. The reduction in the NH flux during autumn and winter implies that less heat is transferred from the ocean to the atmosphere during these seasons over the wind farm areas. In contrast, the heat transfer from the atmosphere to the ocean in spring and summer is less affected. NH fluxes between the atmosphere and ocean decrease due to the decrease in turbulent and radiative fluxes.

The change in the turbulent fluxes is mainly driven by the changes in the near-surface wind speed and TKE. The presence of wind farms reduces the 10 m wind speed by approximately 7% and the TKE by approximately 5% for all wind directions (0—360°), mainly inside and outside the wake downwind of the wind farms. However, near-surface wind acceleration is found upstream of the wind farms due to wind channeling effects. The near-surface wind acceleration effect is more pronounced during spring and summer when atmospheric conditions are generally stable^53,54^. Changes in the turbulent fluxes over the OWF areas strongly depend on seasons, as the wind speed in the North Sea highly varies with seasons. The seasonal change in the LH flux over OWFs is strongest during autumn, which is also reflected in the NH flux, cloud cover, and precipitation (see Fig. SI 7 and SI 8). A reduction in near-surface wind speed and TKE was also observed during the Vertical Enhanced Mixing (VERTEX) field campaign^21^. However, no change in the turbulent fluxes was found in the VERTEX data, which could be because measurements were collected in the wakes of a single wind turbine.

Radiative fluxes are primarily influenced by the increased vertical mixing that transports moisture from atmospheric levels below the rotor area aloft. The uplift and flow convergence increase the moisture flux due to adiabatic cooling in the region over the wind farms. This, in return, increases the amount of low clouds over the wind farms, mainly at their downwind edge. The increase in low clouds results in radiative cooling and an increase in precipitation over the wind farm areas.

Our simulation results indicate that OWFs could potentially impact the sea surface temperature (SST) in the vicinity of OWFs and clusters. The simulated changes in temperature and wind speed are locally limited to approximately 50 km around the OWF clusters. According to the scenario simulations in this study, the slight 2 m temperature increases in OWF areas, and the differences in the annual mean are in the range of 0.05 and 0.25 °C and decrease slightly (mean change less than 0.05 °C) in the mean upwind direction. These changes in 2 m temperature are smaller than the interannual variability (Table 1), which is 1 to 2 orders of magnitude larger. However, the change in the 2 m temperature amplifies the decadal trend over the last 3 decades by approximately 50% (Table 2). Compared to projected climate change impacts at the end of the century in the North Sea region, where changes of 1.7–3.2 °C increase depending on the climate change scenario (NOSCCA^48^) are expected, OWFs would contribute between 5 and 10%. For wind speed, the climate change projections on local to regional scales do not show a consistent direction (NOSCCA^48^). Overall, in this analysis, the impact of large OWFs on precipitation is localized and very small compared to the interannual variability but stronger than the present-day climate change signal (Tables 1 and 2). It is also worth mentioning here that in this analysis, it was found that the impact of OWFs in the North Sea on the land climate is negligible. However, a study based on observational evidence shows that OWFs located very close to the coast (in that case, an onshore observational platform is located 8 and 15 km downwind of the OWFs) can impact the wind speed and precipitation onshore^57^. In our simulations, no wind farms are located close to the coast where their wake effects reach the shore.

The modeled changes in surface climate are significant in the vicinity of the OWF farms and clusters and introduce pronounced spatial structures in the otherwise largely uniform marine climate. However, these scenario simulations provide no evidence that OWF deployment at the scale of EU offshore energy targets has the potential to substantially change the marine and coastal climate on a larger scale.

The results, therefore, suggest that it is important to consider future large-scale clustered offshore wind farms when reconstructing and assessing regional atmospheric dynamics and marine climate, especially when studying future climate change impacts in the marine realm, since the structuring effects on the marine environment are substantial in the vicinity of OWFs. Changes in sea surface winds and heat fluxes impact physical and biogeochemical processes and the local climate in the North Sea^35–38^. OWFs also affect migratory birds and marine animals^58^. Marine ecosystems are structured through hydrodynamics, which is likely influenced by OWFs. Consequently, changes in atmospheric climate due to the impact OWFs must be considered while assessing ecosystem health and fisheries management.

This study is limited to atmosphere-only simulations that lack important air-sea interactions and feedback and, therefore, only provides initial insights into the impact of OWFs on surface climate. Further studies with high-resolution, regional, coupled ocean-wave-atmosphere climate models that include air-sea interactions and feedback processes are required to gain further understanding, assess the impacts of OWFs on the marine climate, test the hypotheses of this study, and explore changes in regional mass and energy budgets arising from accelerated OWF deployment.

## Supplementary Information

Supplementary Information.

## Data Availability

The COSMO-CLM_WF and COSMO-CLM model datasets supporting the results and the COSMO-CLM name lists are available from the authors upon request. The COSMO-CLM simulations employ the community-wide, publicly available (http://www.clm-community.eu) COSMO-CLM code.
