# Supplementary material for: Impacts of accelerating deployment of offshore windfarms on near-surface climate
Source: Sci Rep. 2022 Oct 31;12:18307. doi: 10.1038/s41598-022-22868-9 (PMC9622722; doi:10.1038/s41598-022-22868-9)
Supplement: Supplementary file 1 — Supplementary Information. [file 41598_2022_22868_MOESM1_ESM.docx]

**Supplementary Information**

**Impacts of accelerating deployment of offshore windfarms on near surface climate**

Naveed Akhtar^1^*, Beate Geyer^1^, Corinna Schrum^1,2^

1 Institute of Coastal Systems - Analysis and Modeling, Helmholtz-Zentrum Hereon, Geesthacht, Germany

^2^ Center for Earth System Research and Sustainability, Institute of Oceanography, Universität Hamburg, Hamburg, Germany

*Corresponding author: Naveed Akhtar (naveed.akhtar@hereon.de)

**Supplementary Note 1**

The analysis of the model quality without wind farm effects is shown for the mean annual cycles of the turbulent fluxes, radiative fluxes, 10-m wind speed, 2-m air temperature, 2-m specific humidity, cloud cover, and precipitation for the same period of 2008—2017. It shows good agreement with the observations

For the evaluation of the simulations, we used the following datasets:

- Objectively Analysed Air-sea Fluxes (OAFlux), available from 1958 to present on a 1˚ grid. This dataset contains sea surface fluxes of heat, 10 m wind speed and other variables for the global ocean. It integrates satellite observations with mooring and ship reports to reanalysed surface meteorology using atmospheric models ^1^.
- Surface Radiation Data Set – Heliosat (SARAH) for surface direct shortwave and cloud cover available from 1983 to 2019 on a 0.25˚ grid^2^.
- Advance Very High-Resolution Radiometer (AVHRR) for surface longwave radiation available from 1983 to 2019 on a 0.25˚ grid ^3^
- Global Precipitation Climatology Project (GPCP) for precipitation available from 1979 to present on 0.25˚ grid. This dataset includes various satellite and station datasets ^4^.

Fig. SI. 1. Annual cycle from monthly mean values over the North Sea area, as shown in Fig. 2, in CCLM and observed for (a) 10-m wind speed, (b) 2-m temperature, (c) 2-m specific humidity, (d) total cloud cover, (e) total precipitation, (f) latent heat flux, (g) sensible heat flux, (h) direct downward shortwave radiation, (i) upwelling longwave radiation.

**Supplementary Note 2**

Fig. SI. 2. Annual (first column) and seasonal (columns 2 to 5 for winter, spring, summer, autumn respectively) mean values simulated in CCLM of (first row) 10-m wind speed, (second row) turbulent kinetic energy, (third row) 2-m specific humidity, (fourth row) 2-m temperature, (fifth row) low-l cloud amount, and (sixth row) precipitation outside and inside the wind farms for the prevailing wind directions of 200–280^o^ in the period 2008–2017. This figure was created with Matplotlib (Hunter, J. D., Matplotlib: a 2D graphics environment. Computing in Science and Engineering 9, 2007) and Cartopy (Met office, Cartopy: a cartographic python library with a matplotlib interface. Exeter, Devon, https://scitools.org.uk/cartopy, 2015)

Fig. SI. 3. Annual (first column) and seasonal (columns 2 to 5 for winter, spring, summer, autumn respectively) mean differences (CCLM_WF-CCLM) in (first row) 10-m wind speed, (second row) turbulent kinetic energy, (third row) 2-m specific humidity, (fourth row) 2-m temperature, (fifth row) low-l cloud amount, and (sixth row) precipitation outside and inside the wind farms for the prevailing wind directions of 200–280^o^ in the period 2008–2017. This figure was created with Matplotlib (Hunter, J. D., Matplotlib: a 2D graphics environment. Computing in Science and Engineering 9, 2007) and Cartopy (Met office, Cartopy: a cartographic python library with a matplotlib interface. Exeter, Devon, https://scitools.org.uk/cartopy, 2015)

Fig. SI. 4. As of Fig. SI. 2 but for (first row) net heat (NH) flux, (second row) latent heat (LH) flux,(third row) sensible heat(SH) flux, (fourth row) upwelling longwave (LW) and (fifth row) downwelling shortwave (SW) radiation. This figure was created with Matplotlib (Hunter, J. D., Matplotlib: a 2D graphics environment. Computing in Science and Engineering 9, 2007) and Cartopy (Met office, Cartopy: a cartographic python library with a matplotlib interface. Exeter, Devon, https://scitools.org.uk/cartopy, 2015)

Fig. SI. 5. As of Fig. SI. 3 but for (first row) net heat (NH) flux,(second row) latent heat (LH) flux, (third row) sensible heat(SH) flux, (fourth row) upwelling longwave (LW) and (fifth row) downwelling shortwave (SW) radiation. This figure was created with Matplotlib (Hunter, J. D., Matplotlib: a 2D graphics environment. Computing in Science and Engineering 9, 2007) and Cartopy (Met office, Cartopy: a cartographic python library with a matplotlib interface. Exeter, Devon, https://scitools.org.uk/cartopy, 2015)

Fig. SI.6. Annual mean of downwelling longwave radiation outside and inside the wind farms for all wind directions in the period 2008–2017. This figure was created with Matplotlib (Hunter, J. D., Matplotlib: a 2D graphics environment. Computing in Science and Engineering 9, 2007) and Cartopy (Met office, Cartopy: a cartographic python library with a matplotlib interface. Exeter, Devon, https://scitools.org.uk/cartopy, 2015)

Fig. SI. 7. Transects of the seasonal (coloured, see legend) and yearly mean (dashed gray) for (a) 10-m wind speed, (b) turbulent kinetic energy, (c) 2-m specific humidity, (d) 2-m temperature, (e) low cloud amount, and (f) total precipitation for south-westerly winds (200-280˚) from 2008–2017 taken at transect 2 (German Bight, Fig.2) latitude 54.2 °N–55.6 °N and longitude 5.45 °E–8.0 °E.

Fig. SI.8. Transects of the seasonal (coloured, see legend) and yearly mean (dashed gray) for (a) net heat flux, (b) latent heat, (c) sensible heat, (d) upwelling longwave radiation, and (e) downwelling shortwave radiation for south-westerly winds (200-280˚) from 2008–2017 taken at transect 2 (German Bight, Fig.2) latitude 54.2 °N–55.6 °N and longitude 5.45 °E–8.0 °E.

Fig. SI.9. As of Fig. SI. 4 but for all wind directions (0—360˚). This figure was created with Matplotlib (Hunter, J. D., Matplotlib: a 2D graphics environment. Computing in Science and Engineering 9, 2007) and Cartopy (Met office, Cartopy: a cartographic python library with a matplotlib interface. Exeter, Devon, https://scitools.org.uk/cartopy, 2015)

Fig. SI.10. As of Fig. SI. 3 but for all wind directions (0—360˚). This figure was created with Matplotlib (Hunter, J. D., Matplotlib: a 2D graphics environment. Computing in Science and Engineering 9, 2007) and Cartopy (Met office, Cartopy: a cartographic python library with a matplotlib interface. Exeter, Devon, https://scitools.org.uk/cartopy, 2015)

Fig. SI.11. As of Fig. SI. 2 but for all wind directions (0—360˚). This figure was created with Matplotlib (Hunter, J. D., Matplotlib: a 2D graphics environment. Computing in Science and Engineering 9, 2007) and Cartopy (Met office, Cartopy: a cartographic python library with a matplotlib interface. Exeter, Devon, https://scitools.org.uk/cartopy, 2015)

Fig. SI.12. As of Fig. SI 2 but for all wind directions (0—360˚). This figure was created with Matplotlib (Hunter, J. D., Matplotlib: a 2D graphics environment. Computing in Science and Engineering 9, 2007) and Cartopy (Met office, Cartopy: a cartographic python library with a matplotlib interface. Exeter, Devon, https://scitools.org.uk/cartopy, 2015)

Reference:

1.	Yu, L., Jin, X. & Weller, R. A. Multidecade Global Flux Datasets from the Objectively Analyzed Air-sea Fluxes (OAFlux) Project: Latent and sensible heat fluxes, ocean evaporation, and related surface meteorological variables. *Oa-2008-01* (2008) doi:10.1007/s00382-011-1115-0.

2.	Pfeifroth, U. *et al.* Surface Radiation Data Set - Heliosat (SARAH) - Edition 2. *Satellite Application Facility on Climate Monitoring* (2017).

3.	Karlsson, Karl-Göran; Anttila, Kati; Trentmann, Jörg; Stengel, Martin; Solodovnik, Irina; Meirink, Jan Fokke; Devasthale, Abhay; Kothe, Steffen; Jääskeläinen, Emmihenna; Sedlar, Joseph; Benas, Nikos; van Zadelhoff, Gerd-Jan; Stein, Diana; Finkensieper, St, M. (2020): Surface Radiation Data Set - Heliosat (SARAH) - Edition 2.1. *Journal of Chemical Information and Modeling* **53**, 1689–1699 (2020).

4.	Adler, R. F. *et al.* The version-2 global precipitation climatology project (GPCP) monthly precipitation analysis (1979-present). *Journal of Hydrometeorology* **4**, (2003).
